# A Local Survey of COVID-19: Vaccine Potential Acceptance Rate among Personnel in a Level 1 Trauma Center without Severe COVID-19 Cases

**DOI:** 10.3390/healthcare9121616

**Published:** 2021-11-23

**Authors:** André Nohl, Heithem Ben Abdallah, Veronika Weichert, Sascha Zeiger, Tobias Ohmann, Marcel Dudda

**Affiliations:** 1 Department of Emergency Medicine, BG Klinikum Duisburg, 47249 Duisburg, Germany; sascha.zeiger@bg-klinikum-duisburg.de; 2 Helicopter Emergency Medical Service (HEMS), 47249 Duisburg, Germany; veronika.weichert@bg-klinikum-duisburg.de; 3 Emergency Medical Services, Fire Brigade Oberhausen, 46047 Oberhausen, Germany; 4 Department of Trauma, Hand and Reconstructive Surgery, University Hospital Essen, 45147 Essen, Germany; 5 Research Department, BG Klinikum Duisburg, 47249 Duisburg, Germany; heithem.ben.abdallah@bg-klinikum-duisburg.de (H.B.A.); tobias.ohmann@bg-klinikum-duisburg.de (T.O.); 6 Department of Trauma Surgery, BG Klinikum Duisburg, 47249 Duisburg, Germany; 7 Emergency Medical Services, Fire Brigade Duisburg, 47058 Duisburg, Germany; 8 Emergency Medical Services, Fire Brigade Essen, 45139 Essen, Germany

**Keywords:** COVID-19, vaccination, level 1 trauma, health care workers, public health, survey

## Abstract

Background: Healthcare workers (HCWs) in hospitals are at high risk during the COVID-19 pandemic. Healthcare workers’ infection risk could be amplified during the ongoing pandemic due to various factors, including continuous exposure to patients and inadequate infection control training. Despite the risk healthcare workers face, vaccine hesitancy remains a global challenge. Differences in acceptance rates have ranged from less than 55% (in Russia) to nearly 90% (in China). In order to improve our knowledge of vaccine acceptance and its variation in rates, an evaluation is warranted. A survey was thus administered to healthcare workers. Methods: This survey aimed to address vaccination acceptance among employees in an urban level 1 trauma hospital. It was conducted through a developed and structured questionnaire that was randomly distributed online among the staff (age ≥18 years) to receive their feedback. Results: Among 285 participants (out of 995 employees), 69% were female, and 83.5% were overaged more than 30 years of age. The two largest groups were nurses (32%) and doctors (22%). The majority of respondents reported that they would “like to be vaccinated” (77.4%) and that they trusted the COVID-19 vaccine (62%). Moreover, 67.8% also reported that they felt the vaccination was effective. They reported that vaccination was a method to prevent the spread of COVID-19 (85.15%) and was a way to protect individuals with weak immune systems (78.2%). More importantly, the participants were concerned about other people (80.1%) and believed the vaccine would protect others. On the other hand, the result showed that the majority of participants (95.3%) chose to be vaccinated once everyone else was vaccinated, “I don’t need to get vaccinated”. Results showed that the majority of participants that chose “I don’t need to get vaccinated” did so after everyone else was vaccinated. Our results show that COVID-19 vaccination intention in a level 1 trauma hospital was associated with older age males who are more confident, and also share a collective responsibility, are less complacent, and have fewer constraints. Conclusion: Acceptance of the COVID-19 vaccine is relatively low among healthcare workers (HCWs). Differences in vaccine acceptance have been noted between different categories of HCWs and genders. Therefore, addressing barriers to vaccination acceptance among these HCWs is essential to avoid reluctance to receive the vaccination, but it will be challenging.

## 1. Introduction

COVID-19 is a global pandemic: The 2019 coronavirus (COVID-19) is a potent respiratory virus that causes acute to severe health issues [1]. Various measures have been introduced around the globe to reduce the spread of COVID-19, such as exposure and travel restrictions, referred to as ’lock-downs’. However, the epidemic keeps on spreading despite such efforts [2,3].

### 1.1. Importance of HCWs

Healthcare Workers (HCWs) are the primary victims of COVID-19 and are predisposed to infection [3,4]. Therefore, HCWs are ranked among the groups with the highest risk. The World Health Organization (WHO) has thus considered them for early vaccination along with other front-line workers [5]. HCWs at the level 1 trauma center were trained in the management of COVID-19 patients. Nurses without intensive care experience were trained in interventions for ventilated patients. Non-medical staff were trained in supporting medical procedures. The staff were subjected to strict rules: there was a strict demarcation of the various functional units, staying in shared rooms was forbidden, business trips were prohibited, and in-house training courses were suspended.

Since March 2020, the impact of COVID-19 in the level 1 trauma center has been dealt with by taking strict hygiene measures. These have included mandatory masks, social distancing, bans on professional travel, restrictions on departmental meetings, and prohibited visits by patients’ relatives/attendants. All the patients who were hospitalized or scheduled for hospitalization took a COVID-19 test.

#### 1.1.1. General Knowledge and Attitudes of HCW towards Vaccination

Cultural, sociodemographic, and psychological factors may contribute to vaccination hesitancy. Examining the global impact of COVID-19 vaccine hesitancy and uptake is complicated by the multifaceted nature of this phenomenon [6]. HCWs bridge the gap between healthcare policymakers and patients having a disproportionate influence on patients’ decisions on taking the vaccine. According to earlier studies, the vaccine was perceived differently by different regions in terms of its safety and effectiveness [7,8]. First-world countries located in Northern America and Northern Europe marked the lowest rate with 72–73% of people agreeing that vaccines are safe. However, second- and third-world countries are at risk of vaccination delays due to lack of public trust, shortage of resources, and scarcity of vaccination supply; first-world countries secure a large quantity of the new vaccines without considering the other countries. However, a recent study shows that the COVID-19 acceptance rate is higher (80.3%) among second- and third- world countries compared with Japan (64.6%) and Russia (30.4%) [9].

#### 1.1.2. The Vaccine Hesitancy (VH) among Healthcare Workers

COVID-19 vaccine development and supply remains an ongoing process [10] despite the increasing hesitancy among HCWs in the past decade according to research literature [11,12]. HCWs hesitancy may influence patients’ decisions on taking the vaccine, as they are not likely to recommend it, and/or undermine confidence and contribute to vaccine hesitancy among the general population. For instance, European HCWs have a potential role in determining patient vaccination decision [13], namely France, Verger, and al where vaccination programs in France are threatened by healthcare providers’ reluctance to vaccinate [14]. Meanwhile, the Asian situation, particularly in Singapore, shows that institutional norms and culture may have a powerful influence in setting default behaviors for the hesitancy to take influenza vaccine [15]. On the other hand, two studies indicated that low acceptance rates were also observed among Hong Kong nurses and HCWs in Greece that were willing to take the vaccine as soon as it became available [16,17].

### 1.2. Vaccine

By April 2021, scientists managed to investigate about 82 types of vaccines on humans worldwide. In fact, 23 vaccines reached phase III clinical trials [10,18,19,20,21]. Several vaccines have been authorized in different countries, and vaccinations have begun. In order to facilitate adequate coverage of the COVID-19 vaccine among the general population, understanding community concerns regarding this vaccine is important [22,23]. 

### 1.3. Vaccination Acceptance

Previous studies have demonstrated the degree of vaccine hesitancy among people. However, other studies have been conducted to investigate vaccine acceptance, deducing that its variation is based on a number of factors such as age group, gender, marriage status, educational level, attitudes, and thoughts about COVID-19 infection and immune system ability to fight it [24,25]. The degrees of uncertainty about the vaccine are dissimilar across countries and regions. For instance, the majority of people in poor communities think that the vaccine is safe, and there were 95% in South Asia (94.3% in Malaysia and 91.3% in China) and 94% in East Africa (Ethiopia) who supported it [25,26]. Meanwhile, a lower rate (72–73%) of people in high-income regions, particularly North America and Northern Europe, believe that the vaccine is safe. This low acceptance rate even decreased in Western Europe (59%) and Eastern Europe (50%). The world’s best vaccination model seems to be Israel, since it is vaccinating its population faster than any other country [27], with 111.6 doses administered for every 100 people. Contrariwise, other Middle Eastern countries, such as Kuwait and Jordan, showed the lowest rates of vaccine acceptance at 23.6% and 28.4%, respectively [28].

### 1.4. The Vaccine in Germany

In Germany, 2,689,205 people had been infected with the coronavirus as of 28 March 2021, with 75,708 fatalities. Following the WHO instructions, Germany prepared a program aiming to fight the pandemic, and part of it focused on COVID-19 vaccination. Like many European countries, Germany launched a mass cost-free vaccination program in late December 2020. COVID-19 vaccinations are to begin on December 27 in Germany and the government announced that people over the age of 80 and health care workers will be treated first. Germany government classified three top priority groups: The highest: over 80 years old, HCWs who deal with direct COVID-19 contact patients, a second group with higher priority: those over 70 years old, HCWs who have a higher risk of exposure to COVID-19, essential workers in hospitals; a third group with high priority over 60 years old, the rest of the HCWs who are not included in the first two groups, teachers and daycare workers, retail workers [29]. At the time of our survey, only one vaccine was available for an unpredictable period of time (BioNTech). Furthermore, false reports on the mRNA vaccine are being made, especially via social media, and have led to considerable uncertainty in society. It is almost impossible for medical laypersons to refute these false reports [30,31]. Germany is home to more than 83 million people, while by the end of January 2021, only 1.3 million doses of COVID-19 vaccines were administered. Moreover, 55.8 million doses of the vaccine were ordered initially [32]. However, by the end of March 2021, Germany was the thirty-ninth of all world countries to provide vaccines for its people, and even so, to our knowledge, no studies have been published about HCWs’ acceptance of the COVID-19 vaccine. This study aims to evaluate the willingness of German HCWs to be vaccinated against COVID-19 and the factors that determine that intention in a level 1 trauma hospital.

## 2. Methods

### 2.1. Study Design

A web-based online survey (Umfrageonline.com (accessed on 15 November 2020), enuvo GmbH, Zürich, Switzerland) was conducted among HCWs in BG Klinikum Duisburg, Germany. The study was carried out in a supra-regional trauma center (Level 1 Trauma Center) belonging to the BG Kliniken Group of Hospitals of the German Federal Statutory Accident Insurance (“Deutsche Gesetzliche Unfallversicherung”). HCWs in a level 1 trauma center could be considered as a higher risk of exposure to COVID-19. All participants were voluntary. The survey collected items including demographics data (age, gender, profession). The inclusion criterion for participation in the survey was: age ≥ 18 years, employed clinic staff only (regardless of occupation). Incomplete questionnaires were excluded from the final analysis. The survey link was sent to the employees’ email addresses and a flyer with a QR code was also distributed in all functional areas of the hospital. It was possible to answer the questions on the computer or with one’s own smartphone. Participation was possible from 22 December 2020 to 26 January 2021

### 2.2. 5C Model

To evaluate the vaccination acceptance, we used the 5C model according to Betsch et al. [33]. The 5C model evaluates vaccination acceptance with regard to the following set of issues: confidence, complacency, constraints, calculation, and collective responsibility.

Each of the 5 domains was assessed by a rating point scale (0 = strongly disagree; 100 = strongly agree). Mean scores of items under each domain were computed. A factor analysis using the principal axis factoring approach was conducted to examine the factorial validity of the 5C model in the current population.

### 2.3. Statistics

Statistical analysis was performed using IBM^®^ SPSS^®^ Statistics Version 27.0 (IBM Corporation, Armonk, NY, USA). A *p* < 0.05 level of statistical significance was applied. Chi-Square test of independence was used to determine if there was a significant relationship between the COVID-19 source of information and the occupational groups. A factor analysis using the principal axis factoring approach was conducted to examine the factorial validity of the 5C model in the current population. Multivariate linear and logistic regression models were applied to identify factors associated with COVID-19 vaccine uptake intention.

## 3. Results

### 3.1. Sociodemographic Characteristics

Table 1 represents a summary of the participants’ demographic characteristics. The survey was sent to 620 HCWs. The final sample included 285 HCWs who were eligible for analysis after completing the survey. The split was 68.8% female and 31.2% male. Two hundred and thirty-eight (82.1%) of the participants were older than 30 years, and 43 (16.5%) were younger than 30 years; for the remaining participants (1.4%), no age was reported (Table 1). The majority of respondents were either nurses or doctors (54.1%), and the rest were therapists or personnel with or without medical training (46%).

### 3.2. Survey on Vaccination Readiness

**Figure 1:**
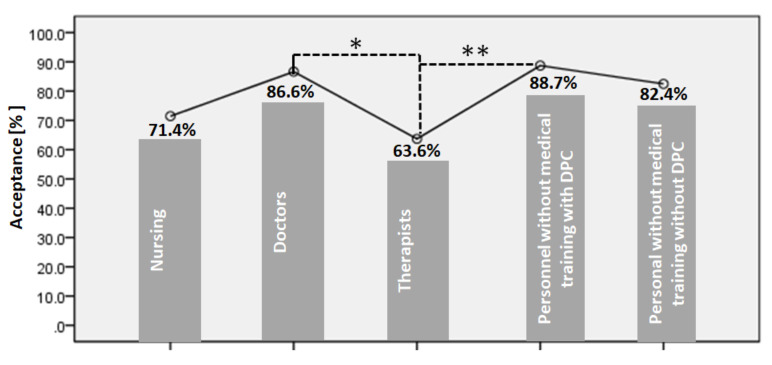
The graph presents the percentage of the potential acceptance of a COVID-19 vaccine among HCWs, *n* = 285, (*p <* 0.05 * and *p <* 0.005 **). DPC direct patient contact.

The first question in our survey addresses the acceptance rate of the COVID-19 vaccine among HCWs. The acceptance rate of the COVID-19 vaccine was 77% among the different categories of HCWs. A significantly higher acceptance rate was shown by both doctors (86.6% (*n* = 64)) and personnel without medical training with DPC (88.7% (*n* = 31)) (*p* < 0.05). In contrast, therapists showed a much lower acceptance rate (63.6% (*n* = 49)), Figure 1.

A sum of 64.8% were confident that the vaccination was effective against COVID-19 but showed a difference (*p* < 0.05) between both genders. Medical workers (74% (*n* = 64)) and personnel without medical training with DPC (73.66% (*n* = 31)) showed a higher level of confidence in taking the vaccine (Table 2). Moreover, we measured a significant difference between groups of various occupations, and remarkably males (74.0%) were more confident compared to females (60.73%) in taking the vaccine (*p* < 0.05). Moreover, only 10.91% of HCWs showed a lower complacency with no significant differences observed among the groups (*p* < 0.05) (Table 2). Our results also show a high calculation value of 83.63%; this means that HCWs evaluate risks of infection and vaccination well before making a decision (Table 2). In addition, the results show that vaccination is a community measure to prevent the spread of COVID-19 (85.15%), and importantly, the participants were concerned about vaccination, and that it could also protect individuals with weak immune systems (78.1%) (Table 2).

### 3.3. Effects of COVID-19 Demands on Vaccination Intention with Work Stress

To assess whether work stress mediated the association between COVID-19-related demands and vaccination intention, we conducted a path analysis with 285 HCWs. The indirect effects of the inconvenience to receive vaccination, beta coefficient (β) = 0.287, *p* < 0.001 and the discomfort with visiting a doctor, β = 0.169, *p* = 0.003, on COVID-19 vaccination intentions with work stress were significant.

### 3.4. Validity of the 5C Model in COVID-19 Vaccine Intention

The 5C model was used (Table 2) to explain participants’ understanding of personal protection against COVID-19. 77.3% of HCWs intended to take the COVID-19 vaccine. Univariate factors associated with stronger intention to take the COVID-19 vaccine were stronger vaccine confidence 0.67 (0.72, 0.6), calculation 0.46 (0.32, 0.73), collective responsibility 0.35 (0.06, 0.54), weaker complacency 0.22 (0.12, 0.30) and constraints 0.28 (0.20, 0.33). The results of Bartlett’s test, χ2 (120) = 1311.82, *p* < 0.001, and the Kaiser–Meyer–Olkin (KMO) measure (0.812) also supported the factorability and sufficiency of the data.

### 3.5. Source of Information of the COVID-19 Pandemic

**Figure 2:**
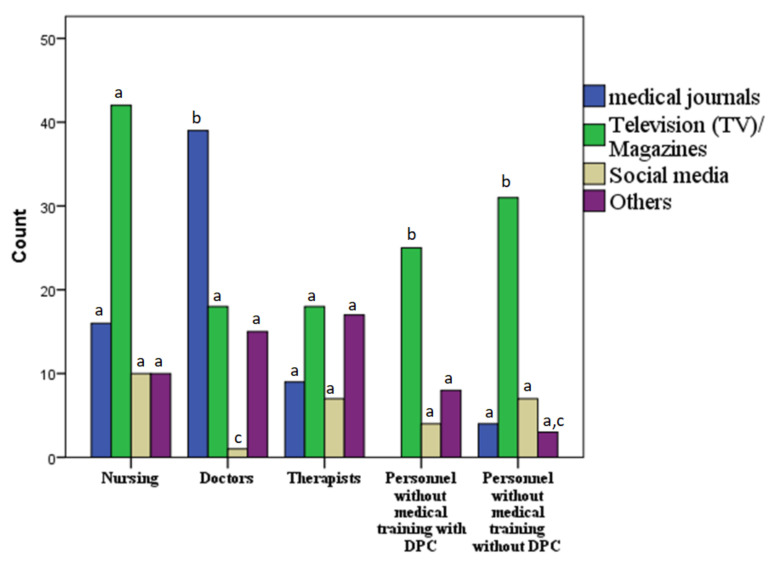
Ways by which participants obtained COVID-19 information, by occupation group. The chi-square test of independence is used to determine if there is a significant relationship between source of information’s and occupations, means with the same letter are not significantly different with *p* ≤ 0.05 according to chi-square test of independence, *n* = 285, (*p* < 0.05). DPC direct patient contact.

Most participants stated television (TV)/magazines (*n* = 134, 47.67%) and medical journals (*n* = 68, 27.15%) as sources of information about COVID-19. The least used sources of information for all five groups were social media and others (*n* = 83, 25.1%) (Figure 2). Significant differences were found in the occupation distribution between the more and less medical education level (*p* < 0.05). Interestingly, a significant difference in occupation was detected within the nursing and the doctors’ groups, since the latter have used the medical journal as a source of information, but nurses mainly relied on TV and newspapers (*p* < 0.05) (Figure 2).

## 4. Discussion

Presently, COVID-19 has become an important topic that concerns people around the globe [34,35]. Thus far, published surveys have primarily focused on the public perception and acceptance of existing vaccines [23]. Therefore, we conducted this survey on HCWs to extend the knowledge and perception of readiness to take a COVID-19 vaccine.

COVID-19 vaccine acceptance: In our survey, 77% of HCWs from 22 December 2020 to 26 January 2021 in a level 1 trauma center reported intention to take the COVID-19 vaccine, with respondents spanning from 63% among (therapists to 71% among nursing professions. The window of time for participation in the survey was prior to the start of the hospital medical staff vaccination campaign. Our results show that the acceptance rate among HCWs was not far from the results observed in China (76.6%), Poland (82%), and French (76.9%) HCWs [36,37,38]. Contrary to our findings, a low level of intention to accept COVID-19 vaccination was observed among HCWs in Turkey (47.6%) and Cyprus (30%), and these could be explained by socio-demographic characteristics in each country [39,40]. A recent study from February 2021 reported a high willingness to take the vaccine at 92% among U.S. HCWs, which is remarkably higher than the rate (77%) in our study [41]. However, the U.S. study made use of an open survey with snowball recruiting, and with no true denominator to establish prevalence. The study is very likely to be biased in this case, since the people who are not interested to take the vaccine may have not responded which makes the actual rate of acceptance lower. In contrast, we report prevalence based on a defined population and actual prevalence. Comparing the vaccine acceptance in terms of gender, our finding was in accordance with the study conducted with U.S. healthcare workers [41].

According to a recent finding concerning emergency medical services in Germany [42], our findings show the acceptance rate to be lower with females than males. These findings might be explained by the fact that males presented a higher rate of mortality than females [43]. Vaccine acceptance was higher among HCWs involved in direct patient care as well as with doctors. The mean acceptance of the vaccine was proportional to increased age. This might be because older people have more fear and consider themselves as being at higher risk to the severity of COVID-19. In our study population, the eldest age group ranging from 50 to 69 showed the highest rate of vaccine acceptance. This was also witnessed in other studies, proving that positive factors of vaccine acceptance include old age and a high education level [44].

Five-C model: Our result using the 5C model revealed that HCWs were confident and ready to take the vaccine but presented weaker constraints. The result from the 5C model revealed that the willingness to be vaccinated related to old age and high education level along with high confidence level, sense of responsibility, and low complacency. The 5C model is intended to make assumptions about the degree of intention to take the vaccine according to the studies conducted on other vaccines [45].

Work stress: Constraints: The COVID-19 related demands during the breakdown were associated with greater work stress, hence a stronger intention to take the COVID-19 vaccine.

COVID-19 source of information: Thus far, published surveys have primarily focused on the public [46,47,48], therefore, we conducted this research to explore the response among HCWs in Germany. Participants of this study used media (e.g., TV/newspaper) most often to obtain information about COVID-19, as well as other sources (medical journals and social media). Other sources, such as employers or family members, were consulted less often for updated information. Similar results were found in other studies that analyzed the sources people used to search for information about COVID-19 [49,50]. In the case of Asian HCWs, the results of news media vs. social media use were compared showing that 39.74% “more often” or “mostly” used news media, while 38.87% “more often” or “mostly” used social media. US HCWs, however, reported a much higher reliance on government websites (66% for clinical decision-makers such as doctors, 54% for other HCW, and 41% for non-HCWs), a much lower reliance on TV news (7% doctors, 17% other HCWs, 29% non-HCWs), and hardly on social media (0% doctors, 2% other HCWs, 2% non-HCWs) [51]. The use of television/magazines by our hospital’s healthcare workers is much higher compared to that among Asian healthcare workers (39.74%) and among U.S. healthcare workers (24%) [51]. They also used social media less (25.1%) than the Asian cohort (38.87%); both relied on social media far more than U.S. healthcare workers (2%) [51]. These differences may be the result of sample methodologies rather than actual practice, but they are of particular concern given that social media does not provide precise reliable knowledge about COVID-19 [52].

Importantly, 82% Of HCWs were older than 30. Therefore, a possible explanation could be that social media apps have become popular and easily accessible, unlike classic sources, for young people. This makes obtaining the information faster and easier. Information about COVID-19 that is published online by official health authorities had a great impact on improving the levels of knowledge among HCWs [50]. Our study revealed that doctors with a higher educational background (graduation or more) and HCWs with direct contact to patients were more aware of the intention and the application of the COVID-19 vaccine. 

### Limitations

There were some limitations to our study. First, this was a cross-sectional study that was conducted online. Second, this study contains self-reported and subjective data; therefore, it may be biased. Third, the small sample size (285) prevented us from receiving sufficient feedback as only a third of the population (995) responded. These limitations do not negate the fact that our findings were valuable, as they present important information on the degrees to which HCWs accept the COVID-19 vaccination. Fourth, work experience was not surveyed. Work experience, in addition to the level of education, can have an influence on the result. 

## 5. Conclusions

Acceptance of the COVID-19 vaccine is relatively low among healthcare workers (HCWs). Differences in vaccine acceptance have been noted between different categories of HCWs and genders. Therefore, addressing barriers to vaccination acceptance among these HCWs is essential to avoid reluctance to receive the vaccination, but it will be challenging. In order to achieve complete control over COVID-19, it would be worthwhile to invest in a multicenter study in Germany following HCWs receiving the COVID-19 vaccine. There should be an implementation of interventions at education and at the policy level, aiming at addressing those issues and promoting COVID-19 immunization programs. Further studies are still required to identify safety and vaccine uptake intention among the general public.

## Figures and Tables

**Table 1:** Demographical characteristics of participants. Impact of COVID-19 acceptance of vaccination of the 285 respondents in the online survey, *n* = 285, (*p <* 0.05).

Items	Respondents (*n* = 285) *n* (%)
**Gender**	
Female	196 (68.8)
Male	89 (31.2)
**Age group in years**	
0–19	1 (0.4)
20–29	46 (16.1)
30–39	67 (23.5)
40–49	59 (20.7)
50–59	83 (29.1)
60–69	25 (8.8)
**Occupation**	
Nursing	90 (31.6)
Doctors	64 (22.5)
Therapists	49 (17.2)
Personnel without medical training with direct patient contact (e.g., reception workers, staff of patient registration, cleaning staff and post workers)	31 (10.9)
Personnel without medical training without direct patient contact (e.g., IT workers, research and finance department and HR department)	51 (17.9)

**Table 2:** 5C model survey according to Betsch et al. [33] to evaluate vaccination hesitancy. STD (standard of deviation), *n* = 285; *p*-value less than *≤* 0.05 is considered significant.

Variables	Count	Percentage (%)	STD	*p*-Value
	(*n*)			
Confidence				
I am completely confident that vaccines are safe	285	62.1	34.1	0.001
Vaccinations are effective	285	67.8	27.02	0.049
Regarding vaccines, I am confident that public authorities decide in the best interest of the community	284	64.6	31.9	0.211
**Complacency**				
Vaccination is unnecessary because vaccine-preventable diseases are not common anymore	284	6.02	16.0	0.035
My immune system is so strong, it also protects me against diseases	284	15. 09	22.06	0.002
Vaccine-preventable diseases are not so severe that I should get vaccinated	284	10. 06	19.07	0.002
**Constraints**				
Everyday stress prevents me from getting vaccinated	284	05. 04	15.06	0.069
For me, it is inconvenient to receive vaccinations	284	11.0	22.0	0.014
Visiting the doctors’ makes me feel uncomfortable; this keeps me from getting vaccinated	284	5.0	15.04	0.05
**Calculation**				
When I think about getting be vaccinated, I weigh benefits and risks to make the best decision possible	284	84.5	24.05	0.004
For each and every vaccination, I closely consider whether it is useful for me	284	80.1	29.05	0.228
It is important for me to fully understand the topic of vaccination, before I get vaccinated	284	86.3	20.09	0.001
**Collective responsibility**				
When everyone is vaccinated, I don’t have to get vaccinated, too	284	69	12.07	0.001
I get vaccinated because I can also protect people with a weaker immune system	284	78.1	33.5	0.30
Vaccination is a collective action to prevent the spread of diseases	284	85.1	25.09	0.001

## Data Availability

The data presented in this study are available on request from the corresponding author.

## Abbreviations

COVID-19	Coronavirus disease 2019
HCWs	Healthcare workers
DPC	Direct patient contact
TV	Television
KMO	Kaiser–Meyer–Olkin
